# An overlooked poultry trade network of the smallholder farms in the border provinces of Thailand, 2021: implications for avian influenza surveillance

**DOI:** 10.3389/fvets.2024.1301513

**Published:** 2024-02-07

**Authors:** Soawapak Hinjoy, Pornchai Thumrin, Jitphanu Sridet, Chat Chaiyaso, Weerachai Suddee, Yupawat Thukngamdee, Oiythip Yasopa, Ong-orn Prasarnphanich, Somruethai Na Nan, Punnarai Smithsuwan, Janjao Rodchangphuen, Carlie L. Sulpizio, Anuwat Wiratsudakul

**Affiliations:** ^1^Office of International Cooperation, Department of Disease Control, Ministry of Public Health, Nonthaburi, Thailand; ^2^Bureau of Disease Control and Veterinary Services, Department of Livestock Development, Ministry of Agriculture and Cooperatives, Bangkok, Thailand; ^3^Division of Epidemiology, Department of Disease Control, Ministry of Public Health, Nonthaburi, Thailand; ^4^Division of Global Health Protection, Global Health Center, US Centers for Disease Control and Prevention, Nonthaburi, Thailand; ^5^Division of Global HIV and TB, Global Health Center, US Centers for Disease Control and Prevention, Nonthaburi, Thailand; ^6^Department of Clinical Sciences and Public Health and the Monitoring and Surveillance Center for Zoonotic Diseases in Wildlife and Exotic Animals, Faculty of Veterinary Science, Mahidol University, Nakhon Pathom, Thailand

**Keywords:** avian influenza, poultry trade network, country border, local community, network analysis

## Abstract

**Introduction:**

In Thailand, community-level poultry trade is conducted on a small-scale involving farmers and traders with many trade networks. Understanding the poultry movements may help identify different activities that farmers and traders might contribute to the spread of avian influenza.

**Methods:**

This study aimed to describe the characteristics of players involved in the poultry trade network at the northeastern border of Thailand using network analysis approaches. Mukdahan and Nakhon Phanom provinces, which border Laos, and Ubon Ratchathani province, which borders both Laos and Cambodia, were selected as survey sites.

**Results:**

Local veterinary officers identified and interviewed 338 poultry farmers and eight poultry traders in 2021. A weighted directed network identified incoming and outgoing movements of where the subdistricts traded chickens. Ninety-nine subdistricts and 181 trade links were captured. A self-looping (trader and consumer in the same subdistrict) feedback was found in 56 of 99 subdistricts. The median distance of the movements was 14.02 km (interquartile range (IQR): 6.04–102.74 km), with a maximum of 823.08 km. Most subdistricts in the network had few poultry trade connections, with a median of 1. They typically connected to 1–5 other subdistricts, most often receiving poultry from 1 to 2.5 subdistricts, and sending to 1–2 subdistricts. The subdistricts with the highest overall and in-degree centrality were located in Mukdahan province, whereas one with the highest out-degree centrality was found in Nakhon Phanom province.

**Discussion:**

The poultry movement pattern observed in this network helps explain how avian influenza could spread over the networks once introduced.

## 1 Introduction

Avian influenza (AI) is an infectious disease caused by type A influenza viruses. AI causes infections in birds, humans, and other mammals such as horses, pigs, and cats (1, 2). Different subtypes of avian influenza viruses have been observed globally (3), and AI viruses (AIVs) are generally not highly contagious to humans (3). However, the first reported evidence of animal-to-human transmission occurred when the highly pathogenic AI (HPAI) A(H5N1) virus was transmitted to humans in the Hong Kong Special Administrative Region in 1997 (4). In late 2003–2004, AI was detected in Thailand and neighboring countries. The Division of Epidemiology, Ministry of Public Health, Thailand, reported 25 human influenza A(H5N1) virus cases, including 17 deaths from 2004 to 2006. The last three persons with confirmed influenza A(H5N1) virus infection reported in Thailand occurred in 2006, and all three died (5).

According to the epidemiological data on the AI A(H5N1) virus, poultry is a primary source of human infection (6). The Thailand Ministry of Agriculture and Cooperative’s Department of Livestock Development (DLD) has implemented various measures to control AI outbreaks and eliminate the disease. Presently, Thailand has had no confirmed reports of AI in poultry in over a decade. However, retrospective data from the World Organization for Animal Health show that AI outbreaks have continued to occur in Thailand’s neighboring countries (7). Human and poultry movements across borders occur on a daily basis. The risk of disease reintroduction is not negligible, and poultry workers may be at greater risk for AI virus infections than the general public due to their daily poultry handling routines. For example, a study conducted in traditional markets in Taiwan suggested that market workers with a higher risk of AI infection seemed to be more careless in preventive behaviors compared to shoppers and those with lower risk (8).

Thailand has a variety of poultry farms, from smallholders to industrialized corporate farms. Thailand’s poultry industry evolved to prioritize exports via vertically integrated companies controlling production chains through contracts. This concentrated system leaves independent smallholders and local markets as a smaller, potentially higher-risk sector for the spread of infectious diseases (9). Moreover, most smallholding farms have few biosecurity precautions, increasing their vulnerability to AI re-emergence and spread (9). Poultry production has increased due to growing export and domestic demand, driven by lower chicken prices (10). Increased consumption and the density of poultry populations could facilitate the spread of AIVs, as animals and farm workers are in close contact (11). An insight into the poultry trade networks is essential to better understanding potential AI transmission sources (12). A comprehension of poultry trade networks and designing surveillance systems can help identify and mitigate the risk of disease spread in poultry production chains, as well as inform more effective AI control measures by understanding local attitudes, knowledge, and beliefs (13, 14).

Social network analysis (SNA) has previously been used to describe how different livestock species are moved and traded in Thailand, including cattle (15), goats (16), pigs (17), and chickens (18). SNA can quantify the structure and geographical distribution of poultry trade networks in Thailand, helping policymakers direct resources to areas at the highest risk of AI transmission. This study explored poultry trade networks in Thailand and how their connectivity facilitates the spread of AI, especially in provinces bordering the Lao People’s Democratic Republic (PDR) and Cambodia. These bordering provinces are at high risk of AI reintroduction as the disease is still prevalent across the borders (7). Focusing data collection on these areas may help strengthen regional AI surveillance.

## 2 Materials and methods

### 2.1 Study sites and target population

The Thailand governmental administrative system is divided into provinces, districts, subdistricts, and villages. In this study, we examined the local poultry trade network within specific subdistricts. The focus was on individuals engaged in trading activities or operating small farms. We herein defined a small or family-run farm as one managing 0.01–0.1 square kilometers of land (19). Smallholder farms in Thailand are characterized by being a part of family livelihood and integrative agriculture (20).

We conducted this study in 99 subdistricts in three districts located in the Nakhon Phanom, Mukdahan, and Ubon Ratchathani provinces (one district in each province). Nakhon Phanom and Mukdahan provinces border Lao PDR, and Ubon Ratchathani province borders both Lao PDR and Cambodia. Study sites located along the borders of Thailand are at high risk of AI outbreaks and are known areas of international poultry movement (21). We selected poultry farmers and poultry traders as the target population. All poultry farmers and traders aged 18 years and older who could speak and read Thai language and had lived in the study area for at least 1 year before participating were eligible. We excluded poultry farmers if they were not included in the Provincial Livestock Offices’ registration database in 2019 and/or had not participated in poultry production for more than 1 month at enrollment. We excluded poultry traders if they had not been selling poultry for more than 1 month at enrollment.

We calculated the sample size of poultry farmers using the formula from the Tool 5 value chain sampling guidelines (22). We estimated the risk of AI infection at 50% (i.e., maximum uncertainty, which yields the largest sample size) of the total poultry farmers (23). We set precision at 7.5% with a z-score of 1.645. We estimated that we needed to sample 112 poultry farmers per district (336 total), with random sampling in each subdistrict. We sampled the farmers based on the 2019 District Livestock Office databases of poultry farmers in the three provinces using Epi Info Version 7 (24). Thai DLD has defined four size categories for poultry farms: backyard (fewer than 3,000 animals), small (3,000–10,000 animals), medium (10,000–50,000 animals), and large (more than 50,000 animals). We studied in rural areas near borders and then only included small-scale poultry farms. Due to the limited number of poultry traders in the study areas, we sampled all poultry traders in each district based on information in the Livestock District Office database. We invited all participants (farmers and traders) through a letter informing them of the requirements and requested they provide written informed consent. With our human ethical approval, we obtained informed consent and conducted in-person interviews with all participants, administered by trained health and veterinary officers using a standardized questionnaire (Supplementary material S1). We collected demographic and risk factor information, including selling or receiving poultry practices from each participant.

### 2.2 Network analysis of the poultry trade network

We built a weighted directed network investigating poultry trading activities across subdistricts in the three study provinces. We defined a *node* as a subdistrict where the participants received and/or sold poultry. A *directed link* was trading activity and direction between the subdistricts. Each link was weighted with the cumulative frequency of the movements addressed by all participants, and a *self-loop* was defined as a link that occurred when both the trader and relevant consumer were identified in the same subdistrict. To identify essential nodes in the network, we measured the degree centrality and betweenness centrality of each node. Degree centrality measures the number of immediate neighbors a node has (25), while betweenness centrality measures how often a node is on the shortest path between two other nodes (26), helping identify the bridging property of each node (27). In degree centrality, the higher the degree centrality of a node, the more connected it is to other nodes in the network. In the context of poultry trade, this means that a node with a high degree centrality has more trading partners. For betweenness centrality, the higher the betweenness centrality of a node, the more important it is to the overall structure of the network. This means that a node with a high betweenness centrality is more likely to be involved in the flow of poultry between different subdistricts.

All network analyses and visualizations were performed with packages “igraph” (28), “dplyr” (29), “maps” (30), “sp” (31), and “leaflet” (32) in program R version 4.1.2 (33).

## 3 Results

### 3.1 Demographic data of participants

We interviewed 346 participants (100% response rate), consisting of 338 poultry farmers (97.7%) and eight poultry traders (2.3%). All poultry traders also reported raising poultry at home. The numbers of women and men were almost equal (184 women and 162 men). Study participants’ ages ranged from 18 to 78 years. The mean and median age was 50 years. Most participants (335; 96.8%) had completed education up to the level of secondary school. The monthly incomes of 254 participants (73.4%) were less than 10,000 Thai Baht (approximately 300 USD). Totally, 120 (34.7%) participants raised mixed-type poultry, which comprised 103 farms with mixed backyard poultry, 54 farms with mixed ducks or geese, and 45 farms with mixed fighting cocks. The remaining 65.3% of poultry farmers raised specialized poultry, with 171, 47, and 8 farms raising only backyard poultry, fighting cocks, and ducks, respectively. In addition, over half (184; 53.2%) raised 2–40 free-range poultry in the household areas together with other animals such as dogs, cats, and cattle during the daytime. At night, these poultry sleep in the coops or small bamboo cages. Overall, 183 (52.9%) participants identified themselves as having over 10 years of experience in poultry farming or trading. There were only 10 participants who had less than 1 year of experience in the poultry sector. Most participants (251; 72.5%) reported having less than 1 h of close contact (feeding and taking care of) with poultry daily.

### 3.2 Poultry trade network

**Figure 1:**
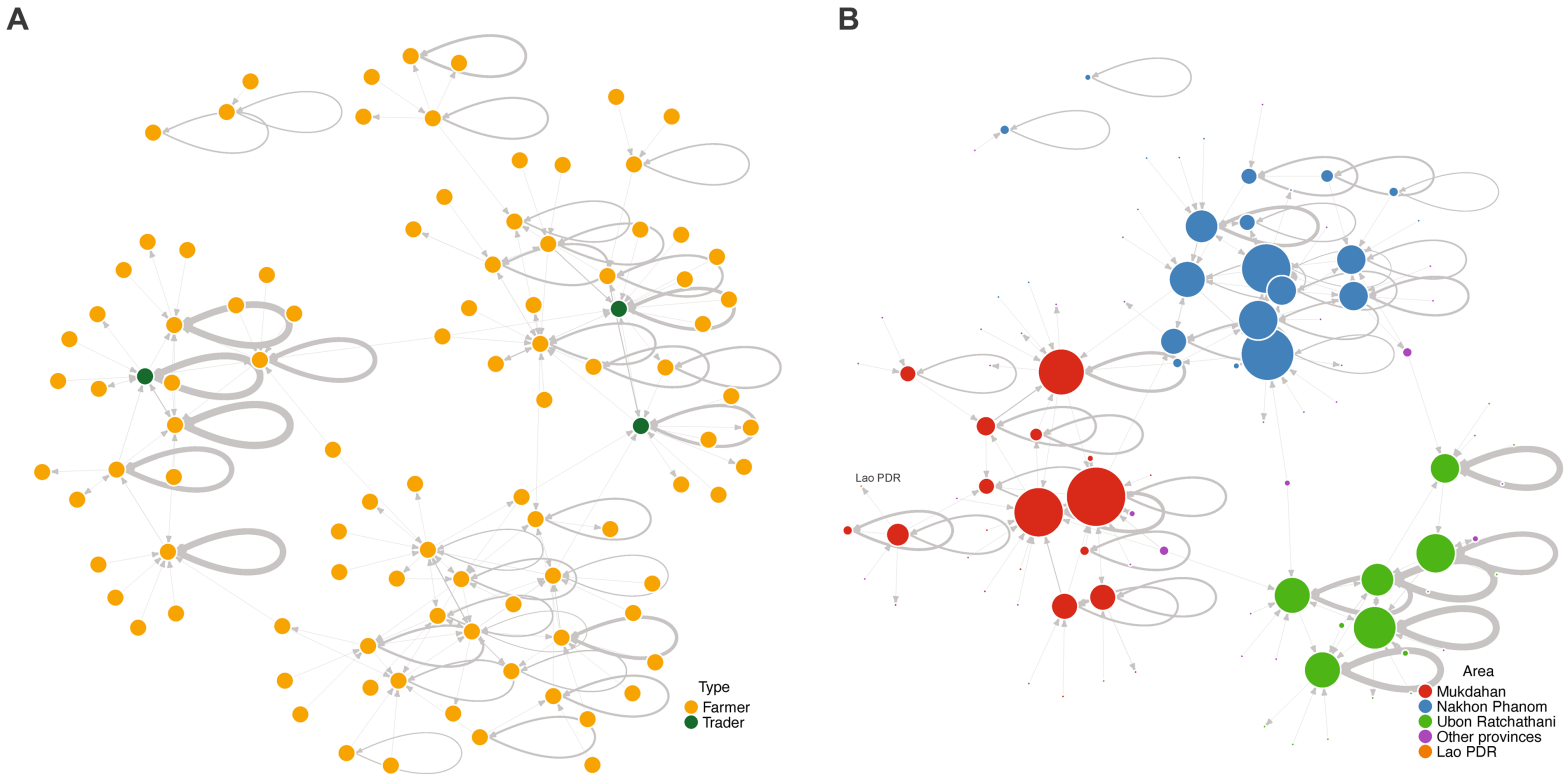
Sociograms of the poultry trade network in the three border provinces—Nakhon Phanom, Mukdahan, and Ubon Ratchathani—of Thailand and other provinces. A *node* refers to a subdistrict where farmers or traders resided at the time of the study, a *directed link* represents the direction of trading activities (buying or selling), and a loop demonstrates self-looping. The thickness of the links indicates the frequency of trading activities. **(A)** Node color shows whether the subdistrict contains only farmers (orange) or farmers and traders (green); **(B)** Node color depicts the provinces or areas where the subdistrict is located. The size of the nodes is proportional to their degree centrality (number of subdistrict trades with the nodes).

The network contained 99 nodes (subdistricts) and 181 links (trading activity between subdistricts). We found that the eight poultry traders identified in our survey lived in three subdistricts of Mukdahan and Ubon Ratchathani provinces (Figure 1A). Among the identified nodes, 28 were located in Nakhon Phanom province. An additional 20, 18, and 32 nodes were found in Mukdahan, Ubon Ratchathani, and other provinces, respectively. Interestingly, only one node was identified outside of Thailand, in Savanna Khet province in Lao PDR (Figure 1B). We found 56 self-loops, representing 56.6% of the nodes in this network.

Overall, in-degree and out-degree centrality values ranged from 1 to 18 (median = 1; interquartile range (IQR) = 1–5), 0–11 (median = 1; IQR = 1–2.5), and 0–8 (median = 1; IQR = 1–2), respectively. Of these, the highest overall and in-degree centralities were found in subdistricts located in Mukdahan province, while the subdistrict with the highest out-degree centrality was identified in Nakhon Phanom province (Table 1). Note that 46 subdistricts did not import poultry from other subdistricts, whereas 13 subdistricts did not export poultry to other subdistricts (detailed results of network measurement in Supplementary material S2).

**Table 1:** Top five centrality values of the subdistricts identified in the poultry trade network in the three border provinces of Thailand.

Province (geocode)	Overall-degree centrality	Province (geocode)	In-degree centrality	Province (geocode)	Out-degree centrality	Province (geocode)	Betweenness centrality
Mukdahan (490108)	18	Mukdahan (490101)	11	Nakhon Phanom (480107)	8	Mukdahan (490108)	1,110
Nakhon Phanom (480107)	16	Mukdahan (490108)	11	Ubon Ratchathani (342505)	7	Nakhon Phanom (480108)	834
Nakhon Phanom (480101)	15	Ubon Ratchathani (342502)	8	Nakhon Phanom (480101)	7	Nakhon Phanom (480112)	818
Mukdahan (490101)	15	Nakhon Phanom (480101)	8	Mukdahan (490108)	7	Mukdahan (490105)	771
Mukdahan (490105)	14	Nakhon Phanom (480107)	8	Ubon Ratchathani (342501)	6	Nakhon Phanom (480115)	711

### 3.3 Geographical distribution of the network

**Figure 2:**
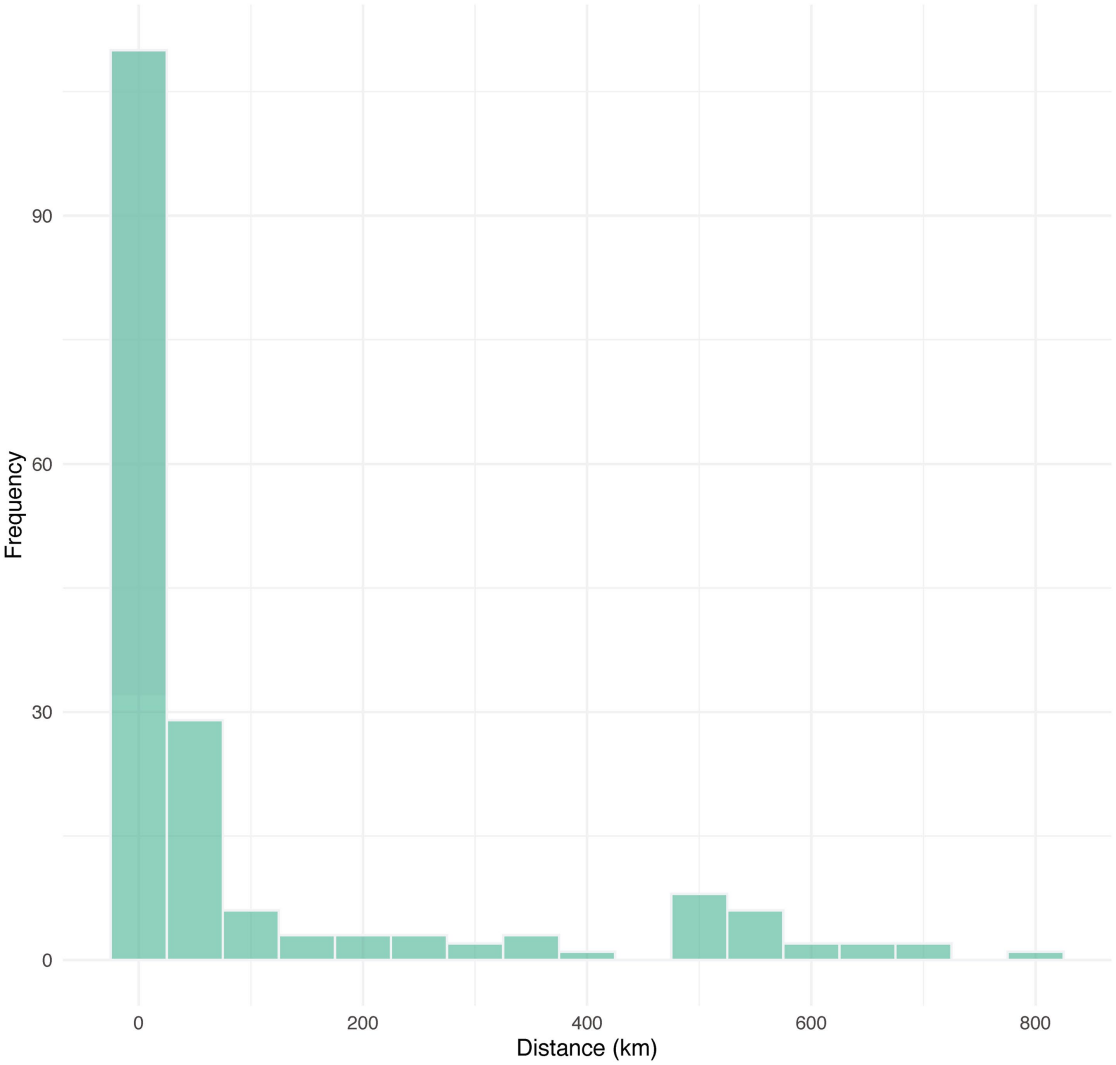
Distance distribution of the poultry movements in the poultry trade network in the three border provinces—Nakhon Phanom, Mukdahan, and Ubon Ratchathani—of Thailand and other provinces.

**Figure 3:**
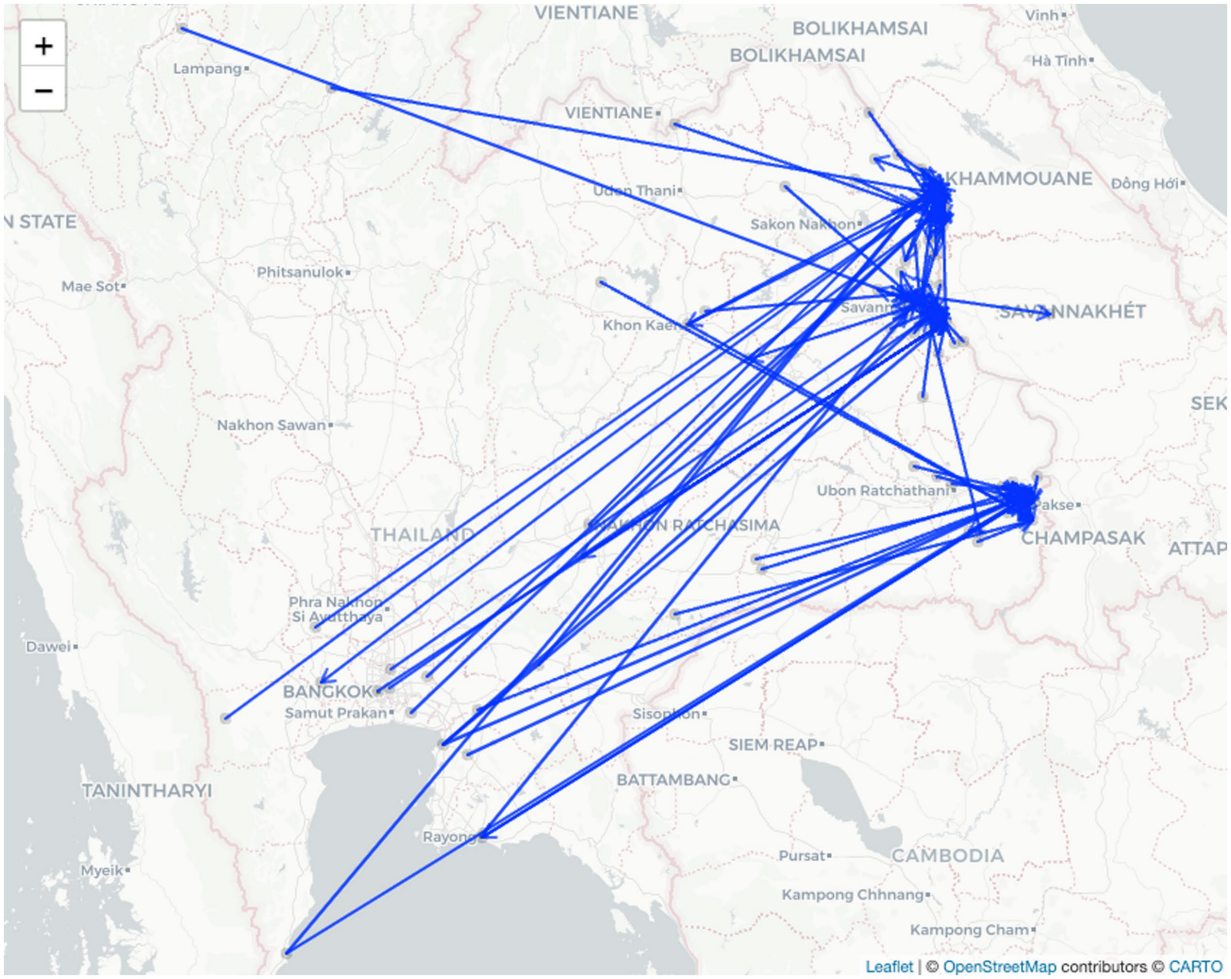
Geographic distribution of the poultry trade network in three Northeastern border provinces—Nakhon Phanom, Mukdahan, and Ubon Ratchathani—of Thailand and other provinces (zoomable version in Supplementary material S3).

The poultry trade network covered 22 provinces, with most trading activity localized in the three participating provinces and their neighbors. Nearly three-quarters (72.3%) of the poultry movements were less than 50 km (Figure 2). The median distance of the movements was 14.02 km, with an IQR of 6.04–102.74 km. However, some traders moved their poultry far from their home locations. For example, poultry could be transported from the northern province of Lamphun to Mukdahan province (645.79 km away) or from the southern province of Prachuap Khiri Khan to Ubon Ratchathani and Mukdahan provinces with distances of 719.63 and 823.08 km, respectively (Figure 3).

## 4 Discussion

In this analysis, we describe the poultry trade network in three Thai border provinces using network measurement parameters. We found the poultry trade in the network to be localized, as evidenced by the self-looping pattern in over half of the nodes involved and that most of the trading activities occurred within each province.

Notably, the subdistricts with multiple trade partners (i.e., with a high degree centrality) were found in the three studied provinces. Network analysis may improve the effectiveness of disease control by focusing primarily on nodes with a high degree centrality (34). Identifying key nodes of trade within the network could help target areas most likely to spread disease and aid in resource allocation decision-making. Strengthening poultry disease surveillance within communities at the subdistrict level and compiling the surveillance data at the provincial level may be the best approach. This surveillance strategy has the capacity to identify and track abnormal events quickly as the key nodes are readily identified with network analysis, allowing authorities to respond efficiently and effectively.

Betweenness centrality can also be used to identify the most influential spreaders of AI and other poultry diseases. It may also be useful in guiding policy decisions to identify areas more accurately in need of assistance in controlling the spread of infection (35). We found that almost three-quarters of the subdistricts included in this analysis did not bridge any trade pairs in the network (betweenness centrality = 0). This result reaffirmed that the poultry trade network in these provinces was distinctly localized. In contrast to those subdistricts with small or zero betweenness centrality values, we identified some subdistricts with high values of betweenness centrality, bridging many trade pairs. AI surveillance programs in these subdistricts could focus efforts on the nodes with high betweenness centrality, designating them as bridges between other nodes and sentinel sites for additional infectious diseases in poultry (36). Further research may identify factors affecting the high betweenness centrality values of these subdistricts to address the risks for relevant local populations.

As previously observed, animal movements can spread infection over long distances (17). Travel restrictions at the local, regional, and national levels are often among the first control policies enforced once a disease outbreak occurs (37). When examined provincially, poultry trade typically occurs only within the province where the trader lives, which is in line with a previous study in central Thailand (18). Our findings clearly showed the self-loops, particularly in Ubon Ratchathani province. In addition, poultry trades in Southeast Asia are often managed informally with little to no documentation, which is a part of local livelihood, as evidenced in previous studies across Thailand (18), Cambodia (38), and Vietnam (39). Rigorous surveillance encompassing these overlooked networks is critical to reducing the risk of AI transmission. Most of the participants in our study were older and had limited education and income, which may have limited their ability to trade outside their provinces. This pattern of poultry movement in the regions may reduce the risk of disease transmission. An additional study on the impacts of socioeconomic factors on poultry trade may provide insights into this aspect of the trade network. Indeed, we used our questionnaire results to investigate the impact of certain socioeconomic factors on risk perception in one of our prior studies (40). However, there was still some long-distance poultry trade, and our findings were consistent with previous studies on livestock movements in Thailand, which showed that most of the movements were managed locally with some remote translocations (15, 16). Future studies on long-distance movements of poultry trading may provide insight into trader motivations and their impacts on AI transmission. The central region of Thailand was the most frequent destination in the poultry trade beyond the three noted provinces. Approximately half of the country’s chickens are raised in central Thailand, a relatively small but densely populated region, and most chicken exports come from farms in the region (41). Strengthening poultry disease surveillance and real-time information sharing across regions could be enhanced to improve early disease detection, especially in Thailand’s central and northeastern regions.

In March 2021, Lao PDR reported that sentinel surveillance identified the first human AI A(H5N6) infection outside China (42). Afterward, an animal investigation in Lao PDR detected Muscovy ducks testing positive for the same AI subtype (42). Genetically, the virus originated from the reassortment of AI A(H5N1) and AI A(H6N6), which extensively circulates in ducks in China (43). In order to prevent the spread of AI and emerging infectious diseases in this region, integrated active surveillance with multi-sectoral collaboration that uses a One Health approach to balance the health of people, animals, and the environment (44) could focus on risk areas identified through social network analysis, especially along countries’ borders. At the time of data collection, Thailand and other countries in the region were facing an increase in COVID-19 infections, which sparked regional border closures. Despite travel restrictions in the region, we identified a trade network outside the country in Savanna Khet province in Lao PDR. Nevertheless, the Thai government lifted lockdown measures to cope with the COVID-19 pandemic almost a year prior to our study. Human and animal movements were proceeding normally within the country. However, border entry points and animal quarantine facilities were not operating as usual. Therefore, only one node outside Thailand was identified. Regardless, our questionnaire was designed to ask poultry farmers about their usual practices in the past, not just the current situation. This network demonstrates that despite the rising number of COVID-19 infections in the area and border closure mandates, small-scale international trade continued to occur but was less frequent than in pre-COVID-19 conditions. Human movement across the Thai-Myanmar border decreased substantially during regional COVID-19 border closures; however, movement between the two countries quickly rebounded after the relaxation of COVID-19 border closure policies (45). We expect the international poultry trade network to exhibit similar patterns at our study sites. A monitoring system for such dynamics could be implemented to enhance the cross-bordered surveillance system for AI. In addition, a recent review article suggested that AI was found more frequently and widely spread globally than it was before. Of those, almost half were classified as highly pathogenic AI A(H5N1) (46). Global surveillance of AI infections in both humans and animals should be rigorously maintained and strengthened, as evidenced in the present study, which shows that poultry trades around the border areas were still traditionally managed.

Limitations of this study include the impact of COVID-19 on study participants’ typical trading and movement behaviors. For example, animal control points and international live bird markets were temporarily closed by order of the Thailand provincial Communicable Disease Committee to halt transmission of COVID-19 and, therefore, could not be included in our study. Additional data on the trade across borders could be collected post-pandemic to complete the whole network. Nevertheless, this study allowed us to observe the local poultry trade network under unusual circumstances, even though some unregistered producers were left out. Additionally, our study was totally carried out during the time of the COVID-19 pandemic. Hence, we could not directly compare our results with the non-COVID-19 situations. Our study strictly focuses on small-scale poultry networks; thus, large industrial farms were not included. A future network study covering all sectors could help identify different sectors’ risks. Moreover, only three provinces were included in this study. Poultry farms in these three provinces accounted for only 6.25% of all poultry farms in Thailand (173,901/2,783,457 farms) in 2021 (47). A future study extending to cover a wider geographical area is suggested, as AI has not been identified in Thailand for over a decade, even though AI surveillance activities have continuously been performed. Without any prevalence data, we need to maximize our sample size as calculated in the methods. In this study, we built a static network to describe the local poultry trade network cross-sectionally. A longitudinal data collection could be helpful in the future to capture additional changes over time. Finally, we holistically analyzed the network at the subdistrict level to describe the poultry trade patterns in the study area. A better insight into the demographic characteristics of the participants involved was previously addressed in our previous study (40).

Our findings indicate that the poultry trade in three border provinces of Thailand was relatively localized, as revealed by multiple self-loops. We identified subdistricts with high centrality values that reflect the substantial movement activities throughout the different study areas. Insights into the Thai poultry trade network provided by the SNA have important implications for identifying areas vulnerable to the re-emergence and spread of AI. Implementing a strengthened surveillance system with control measures in areas with extensive poultry trading could help mitigate the transmission of AI. Furthermore, SNA can greatly enhance risk communication and biosecurity measures, which can help to reduce the spread of disease across the entire value chain. SNA allows for targeted strategies, making disease risk reduction more effective and efficient.

## Data availability statement

The original contributions presented in the study are included in the article/Supplementary material, further inquiries can be directed to the corresponding author.

## Ethics statement

The studies involving humans were approved by Research in Human Subjects, Department of Disease Control, Ministry of Public Health, Thailand. The studies were conducted in accordance with the local legislation and institutional requirements. The participants provided their written informed consent to participate in this study.

## Author contributions

SH: Conceptualization, Data curation, Formal analysis, Funding acquisition, Investigation, Methodology, Project administration, Resources, Validation, Writing – original draft, Writing – review & editing, Supervision. PT: Conceptualization, Data curation, Investigation, Methodology, Project administration, Resources, Writing – review & editing. JS: Data curation, Investigation, Resources, Writing – review & editing. CC: Data curation, Investigation, Resources, Writing – review & editing. WS: Data curation, Investigation, Resources, Writing – review & editing. YT: Data curation, Investigation, Resources, Writing – review & editing. OY: Data curation, Investigation, Resources, Writing – review & editing. OP: Funding acquisition, Investigation, Project administration, Writing – review & editing. SN: Funding acquisition, Investigation, Project administration, Writing – review & editing. PS: Data curation, Investigation, Resources, Writing – review & editing. JR: Data curation, Investigation, Resources, Writing – review & editing. CS: Validation, Writing – review & editing. AW: Conceptualization, Data curation, Formal analysis, Investigation, Methodology, Software, Supervision, Validation, Visualization, Writing – original draft, Writing – review & editing.
