# Supplementary material for: An overlooked poultry trade network of the smallholder farms in the border provinces of Thailand, 2021: implications for avian influenza surveillance
Source: Front Vet Sci. 2024 Feb 7;11:1301513. doi: 10.3389/fvets.2024.1301513 (PMC10879335; doi:10.3389/fvets.2024.1301513)
Supplement: Supplementary file 2 [file Data_Sheet_1.PDF]

The Poultry Trade Network Questionnaire for Poultry Farmers

Section 1 General information

Age................Year   Gender ☐ Male   ☐ Female

Status   ☐ single   ☐ married   ☐ divorce

Education level ☐ Under bachelor   ☐ Bachelor   ☐ Master   ☐ Doctorate

Average monthly income (Baht)

☐ ≤10,000   ☐ 10,001 – 20,000   ☐ 20,001 – 30,000   ☐ 30,001 – 40,000   ☐ >40,000 บาท

Types of farmed poultry

☐ Broiler   ☐ Layer chicken   ☐ Native chicken   ☐ Fighting cock   ☐ Meat Duck

☐ Egg Duck   ☐ Goose   ☐ Quail   ☐ Other please identify............................

Total number of poultry in the farm ....

Experience of poultry farming (Year)   ☐ ≤1   ☐ 1 – 5   ☐ 5 – 10   ☐ >10

Time spent on poultry production per day (Hour)   ☐ ≤1   ☐ 1 – 3   ☐ 3 – 5   ☐ 5 – 8

Length of stay in the area (Year)   ☐ <1   ☐ ≥1

Poultry intake information (please indicate subdistrict)

1. Location..District......................................Province.......................................

Poultry type...........................Average (Number per time)........................Frequency (Time per year)......................

2. Location..District......................................Province.......................................

Poultry type...........................Average (Number per time)........................Frequency (Time per year)......................

3. Location..District......................................Province.......................................

Poultry type...........................Average (Number per time)........................Frequency (Time per year)......................

4. Location..District......................................Province.......................................

Poultry type...........................Average (Number per time)........................Frequency (Time per year)......................

5. Location..District......................................Province.......................................

Poultry type...........................Average (Number per time)........................Frequency (Time per year)......................

Poultry sales details (Please indicate subdistrict)

1. Location..District......................................Province.......................................

Poultry type...........................Average (Number per time)........................Frequency (Time per year)......................

2. Location..District......................................Province.......................................

Poultry type...........................Average (Number per time)........................Frequency (Time per year)......................

3. Location..District......................................Province.......................................

Poultry type...........................Average (Number per time)........................Frequency (Time per year)......................

4. Location..District......................................Province.......................................

Poultry type............................Average (Number per time).........................Frequency (Time per year).......................

5. Location...District.......................................Province..

Poultry type............................Average (Number per time).........................Frequency (Time per year).......................

The Poultry Trade Network Questionnaire for Poultry Traders

Section 1 General information

Age................Year Gender ☐ Male ☐ Female

Status ☐ single ☐ married ☐ divorce

Education level ☐ Under bachelor ☐ Bachelor ☐ Master ☐ Doctorate

Average monthly income (Baht)

☐ ≤10,000 ☐ 10,001 – 20,000 ☐ 20,001 – 30,000 ☐ 30,001 – 40,000 ☐ >40,000 บาท

Types of poultry sales

☐ Broiler ☐ Layer chicken ☐ Native chicken ☐ Fighting cock ☐ Meat Duck

☐ Egg Duck ☐ Goose ☐ Quail ☐ Other please identify............................

Experience of poultry sales (Year) ☐ ≤1 ☐ 1 – 5 ☐ 5 – 10 ☐ >10

Time spent on poultry sales per day (Hour) ☐ ≤1 ☐ 1 – 3 ☐ 3 – 5 ☐ 5 – 8

Length of stay in the area (Year) ☐ <1 ☐ ≥1

Poultry Purchase Details (please indicate subdistrict)

1. Location..District......................................Province..

Poultry type..........................Average (Number per time)........................Frequency (Time per year)......................

2. Location..District......................................Province..

Poultry type..........................Average (Number per time)........................Frequency (Time per year)......................

3. Location..District......................................Province..

Poultry type..........................Average (Number per time)........................Frequency (Time per year)......................

4. Location..District......................................Province..

Poultry type..........................Average (Number per time)........................Frequency (Time per year)......................

5. Location..District......................................Province..

Poultry type..........................Average (Number per time)........................Frequency (Time per year)......................

Poultry Sales Details (please indicate subdistrict)

1. Location..District......................................Province..

Poultry type..........................Average (Number per time)........................Frequency (Time per year)......................

2. Location..District......................................Province..

Poultry type..........................Average (Number per time)........................Frequency (Time per year)......................

3. Location..District......................................Province..

Poultry type..........................Average (Number per time)........................Frequency (Time per year)......................

4. Location..District......................................Province..

Poultry type..........................Average (Number per time)........................Frequency (Time per year)......................

5. Location..District.......................................Province..

Poultry type............................Average (Number per time).........................Frequency (Time per year).......................
